# Machine Learning Algorithms for Prediction of Survival by Stress Echocardiography in Chronic Coronary Syndromes

**DOI:** 10.3390/jpm12091523

**Published:** 2022-09-16

**Authors:** Lauro Cortigiani, Danila Azzolina, Quirino Ciampi, Giulia Lorenzoni, Nicola Gaibazzi, Fausto Rigo, Sonia Gherardi, Francesco Bovenzi, Dario Gregori, Eugenio Picano

**Affiliations:** 1 Ospedale San Luca, Azienda Usl Toscana Nord Ovest, 55100 Lucca, Italy; 2 Biostatistics, Epidemiology and Public Health Unit, Padova University, 35126 Padova, Italy; 3 Department of Environmental and Preventive Science, University of Ferrara, 44124 Ferrara, Italy; 4 Cardiology Division, Fatebenefratelli Hospital, 82100 Benevento, Italy; 5 Department of Cardiology, Parma University Hospital, 43125 Parma, Italy; 6 Cardiology Division, Villa Salus Hospital, Mestre, 30174 Venezia, Italy; 7 Cardiology Division, Cesena Hospital, 47521 Cesena, Italy; 8 CNR, Institute of Clinical Physiology, Biomedicine Department, 56100 Pisa, Italy

**Keywords:** stress echocardiography, flow velocity reserve, machine learning, random forest, survival

## Abstract

Stress echocardiography (SE) is based on regional wall motion abnormalities and coronary flow velocity reserve (CFVR). Their independent prognostic capabilities could be better studied with a machine learning (ML) approach. The study aims to assess the SE outcome data by conducting an analysis with an ML approach. We included 6881 prospectively recruited and retrospectively analyzed patients with suspected (*n* = 4279) or known (*n* = 2602) coronary artery disease submitted to clinically driven dipyridamole SE. The outcome measure was all-cause death. A random forest survival model was implemented to model the survival function according to the patient’s characteristics; 1002 patients recruited by a single, independent center formed the external validation cohort. During a median follow-up of 3.4 years (IQR 1.6–7.5), 814 (12%) patients died. The mortality risk was higher for patients aged >60 years, with a resting ejection fraction < 60%, resting WMSI, positive stress-rest WMSI scores, and CFVR < 3.The C-index performance was 0.79 in the internal and 0.81 in the external validation data set. Survival functions for individual patients were easily obtained with an open access web app. An ML approach can be fruitfully applied to outcome data obtained with SE. Survival showed a constantly increasing relationship with a CFVR < 3.0 and stress-rest wall motion score index > Since processing is largely automated, this approach can be easily scaled to larger and more comprehensive data sets to further refine stratification, guide therapy and be ultimately adopted as an open-source online decision tool.

## 1. Introduction

Stress echocardiography (SE) based on regional wall motion abnormalities (RWMAs) is embedded in clinical practice and general cardiology guidelines for patients with chronic coronary syndromes. Recently, the standard methodology of SE based on RWMAs was enriched by the coronary flow velocity reserve (CFVR) in the mid-distal left anterior descending coronary artery (LAD), which was obtained with a pulsed-wave Doppler during vasodilator SE [1,2]. The RWMA detects hemodynamically significant epicardial coronary artery stenosis, and the CFVR assesses the impairment of coronary microvascular circulation. They recognize different and complementary pathophysiological targets, and show independent and incremental value in predicting survival [3].

The information on outcome prediction obtained by SE should be transferred to the clinical end-user through intuitive informatic interfaces for immediate impact at the bedside. In addition, the conventional, time-honored Cox analysis [4] may miss more subtle information on the interaction of variables, which can be potentially unmasked by the machine learning (ML) approach [5,6]. An approach based on artificial intelligence has the recognized potential to identify unsuspected patterns [7], and this can be especially relevant in the field of risk stratification by cardiac functional stress testing due to multiple parameters used in comprehensive stress testing and the variable weight of covariates. In this way, outcome data can be made readily available for clinical use [8]. The data set of large-scale, prospective, multicenter SE studies is ideally suited for this analysis since patients were studied with the same methodology by accredited centers with extensive experience in joint reading for decades [9]. The present study aims to detect, via the ML approach, information and prognostic relationships between RWMAs, CFVR, and survival. Moreover, the developed model has been transferred to a user-friendly predictive tool in a web application for the clinical end-user.

## 2. Materials and Methods

**Figure 1:**
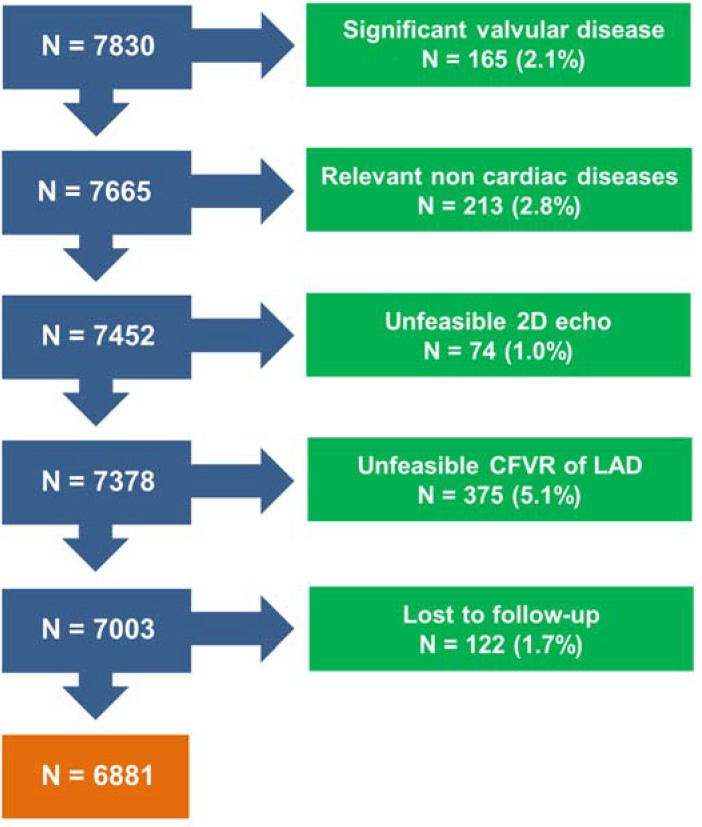
Consort diagram. Flow diagram showing how many individuals were excluded at each exclusion step.

The study population was made up of 6881 prospectively recruited and retrospectively analyzed patients (4080 men, 2801 women; median age 67 years, interquartile range (IQR) 59–74) with suspected (*n* = 4279) or known (*n* = 2602) coronary artery disease who were enrolled in 5 Italian centers, after methodology standardization and upstream quality control of certified readers, at different times: Lucca from 2005 to 2019, Mestre from 2003 to 2010, Cesena and Pisa from 2005 to 2011, and Benevento from 2011 to 2019. We already reported, with a shorter follow-up, 4313 patients in prognostic studies on CFVR and RWMAs [9]. The external validation cohort was made up of an additional 1002 patients recruited by a single, independent center (Parma) in the same time period (2008–2011). Exclusion criteria were hemodynamically significant valvular disease, prognostically relevant noncardiac diseases (cancer, end-stage renal disease, or severe obstructive pulmonary disease), or a suboptimal acoustic window precluding satisfactory imaging of the left ventricle (for 2D echo) or flow Doppler (for CFVR assessment of LAD). In addition, 122 patients were lost to follow-up. Accordingly, of the initial 7830 patients arriving to the SE lab for testing, 949 were excluded (Figure 1), and the remaining 6881 patients formed the study group.

All patients underwent dipyridamole (0.84 mg/kg in 6 min) SE with the dual assessment of RWMAs (wall motion score index (WMSI), each segment scored from 1 to 4, 17 segment models of left ventricle) and CFVR (peak/rest ratio of maximal diastolic flow velocity) of LAD. The positivity cutoff value for the RWMA diagnostic of myocardial ischemia was a WMSI peak > rest for ≥0.12, equivalent to stress-induced worsening of at least 1 grade in ≥2 segments. Myocardial viability was considered present when at least two adjacent segments of the same vascular territory of the left ventricle with resting dysfunction (severe hypokinesia or akinesia) showed a decrease of at least one point of the segmental score during SE. Follow-up information was available in all.

All-cause mortality was the only end-point. Patients undergoing coronary revascularization were not censored at the time of the procedure. Risk factors were defined according to standard recommendations [10].

The study protocol was reviewed and approved by the Comitato Etico Lazio-1 on 16 July 2016; it was published on ClinicalTrials.gov with identifier NCT 030.49995 as a part of the multicenter echo international study network and, starting 2016 with an unchanged methodology, as a part of the SE 2020 study. Written informed consent was obtained from all patients before testing.

### Statistical Analysis

Descriptive statistics. Descriptive statistics are reported according to the predictors and stratified follow-up survival status. Twenty-one survival predictors were considered for the analysis: age, gender, family history of coronary artery disease, cigarette smoking, diabetes mellitus, hypertension, hypercholesterolemia, left bundle branch block, prior myocardial infarction, prior coronary artery bypass grafting (CABG), prior percutaneous coronary intervention (PCI), ongoing anti-ischemic therapy, beta blocker, calcium antagonist, nitrate, resting left ventricular ejection fraction (LVEF), resting WMSI, stress WMSI, delta WMSI, inducible ischemia, and CFVR of LAD. 

Continuous data were synthesized as medians (I, III quartiles), whereas the categorical data are reported as a percentage and absolute frequencies. As a benchmark model, both univariable and multivariable Cox regression hazard estimates are reported with 95% confidence intervals and *p*-values.

Random forest algorithm. A random forest (RF) survival model was implemented to predict the survival function according to the patient’s characteristics.

The RF algorithm was selected because it represents a promising method for the identification of the variables associated with time-to-event outcomes in complex data. The RF provides similar estimates in comparison with the commonly used Cox regression analysis, but also addresses the problem of multicollinearity for the data. Moreover, the partial dependency plots produced via RF model estimation are useful in investigating the direction and potential nonlinearity of associations by translating them into clinically understandable associations [11].

The RF [12] is a nonparametric ML algorithm not based on distributional or functional assumptions concerning the relationship of covariates to the response variable. The method is an ensemble learning tool developed for classification, regression, and other predictive tasks that operate by constructing a forest of decision trees at training time. The RF output is the mode of the classes (for classification task) or the average prediction (for a regression task) of the individual decision trees [13]. A single decision tree is a (classification or regression) ML predictive tool that could be trained by performing repeated splitting procedures on the data. This process is repeated, on each derived subset, in a recursive manner (recursive partitioning). The recursion is completed when the subset splitting procedure no longer adds value to the prediction performance [14].

The RF survival [15,16] is an extension of Breiman’s RF techniques applied to survival data, allowing efficient nonparametric analysis of time-to-event data. Of note, constructing an RF model from base learners such as a decision tree can significantly improve the learning performance [17].

The algorithm was tuned to identify the optimal number of variables available for splitting the decision tree at each tree node (mtry) and node size parameter for an RF model by minimizing an out-of-bag error metric computed in terms of the C-index on the survival outcome. Main importance measures are detailed in the Supplementary Materials (Importance Measures, Supplementary Material S1). In summary, the variable importance (VIMP) [15] value close to zero indicates that the variable does not contribute to the predictive accuracy of the model. Minimal depth [18] is a proxy of the feature predictiveness, and a smaller measure indicates a higher impact of the variable on the RF prediction. The variable dependence plot [19] shows the predicted response as a function of the variable of interest, with each predicted point dependent on the full combination of all other covariates, not only on the covariate of interest. The partial dependence plot [20] area is a risk-adjusted alternative to variable dependence. The minimal depth interaction effect was computed across features. The minimal depth measure is computed as the average of the depth of the variable *i* relative to the root terminal node. The interaction depth between variables *i* and *j* were computed by considering the minimal depth of the variable *j* concerning the maximal subtree for the variable *i* [18].

The validation process was conducted (A) during the RF training phase; (B) by performing a 10-fold cross-validation on the data; (C) by conducting an external validation on an external study cohort (Supplementary Material S2, Table S1). Procedure (A) and (B) are internal validations; procedure (C) is an external validation.

(A) The validation conducted during the RF tuning resampling runs was conducted as follows:
  - Each of the 500 trees composing the RF algorithm was developed by using a different bootstrap random sample of the data (training-set data) that was 0.632 times the entire study size.
  - The out-of-basket (OOB) sample excluded during the construction of the single tree served as the test set by deriving the prediction for each observation.
  - Based on the prediction derived from the single trees, survival curves for OOB patients were calculated.
  - For each subject, the average survival curves across 500 runs were calculated to be considered the subject’s final survival.
  - At the end of the runs, the OOB C-index performance (perfect prediction = 1) was computed by comparing the true survival with the average OOB survival.
(B) The RF validation was also carried out by considering a 10-fold cross-validation (CV) procedure. The procedure consists of the subdivision of the total data set in 10 parts of equal sample size and, at every step, the 10th part of the data set becomes the validation part, whereas the remaining part constitutes the training set. The predictive tool is trained for each of the 10th parts, avoiding, therefore, problems of overfitting, but also of asymmetrical sampling (and, therefore, those affected by distortion) of the observed sample, which is typical of the subdivision of the data in only two parts (that is training/validation) [21,22]. The performance was assessed by reporting the Harrel C-index statistics. The internal validation performance was calculated also, for comparative purposes, for the conditional tree (CTree [23]), gradient boosting machine (GBM [20]), elastic net regularized Cox regression (Coxnet [24]), and extreme boosting machine (Xgboost [25]).
(C) RF external validation: The RF predictive tool was also externally validated on a cohort of 1002 patients (the external study cohort details are reported in Table S2 of the Supplementary Materials). The RF predictions were calculated on the external cohort and compared with the observed survival of the external cohort data by calculating the C-index concordance measure.

The validation procedures B and C were performed for comparative purposes, as was a classical Cox regression model approach. We followed the proposed Requirements for Cardiovascular Imaging-Related Machine Learning Evaluation (PRIME) checklist [26], as reported in the Supplementary Material S3.

A web application was developed to predict the survival function according to the patients’ characteristics (https://r-ubesp.dctv.unipd.it/shiny/Dynamic/, accessed on 3 May 2022). The app allows the survival functions to be constructed dynamically; the curves can be added, for each new patient profile, to the same plot within the same session.

The Shiny Web application was developed by providing different sub-panels:

- The survival curve panel is the main application section where the survival prediction is plotted according to the patient’s features selected on the left side of the webpage. The web app includes multiple patients’ profile predictions, enabling us to compare the survivals for the selected patient characteristics.
- The identified patient profiles are stored in the patient’s profile panel.
- The variable importance depth measure plot is reported in the variable importance section of the web application.
- The marginal effect plots are represented in the marginal effect section for the four leading predictors according to the minimal depth of a variable measure. The explanation of the basic issues concerning the RF algorithm is indicated in the random forest section.

Computations were performed by using R 3.4.2 (R Foundation for Statistical Computing, Vienna, Austria) with the rfsrc [15] and ggRandomForests [27] packages.

## 3. Results

Descriptive statistics. SE was positive for myocardial ischemia in 678 patients (Table 1). The median CFVR of LAD was 2.3 (IQR 2.0–2.6) (Table 1).

During a median duration of follow-up of 3.4 years (IQR 1.6–7.5), 814 (12%) patients died. In total, 954 subjects (14%) underwent coronary revascularization (205 CABG, 741 PCI) after a median of 134 days (IQR 12–330) from SE and were not censored.

In the external validation cohort, the median duration of follow-up was 8.3 years (IQR 6.7–79.1) and 161 (16%) deaths occurred.

All the predictors were significantly associated with the survival outcome, as indicated in the Cox univariable analysis results, except cigarette smoking and prior PCI (Table 1). Multivariable predictors of mortality were age (HR 1.08, 95% CI 1.06–1.09; *p* < 0.0001), CFVR of LAD (HR 0.59, 95% CI 0.46–0.75; *p* < 0.0001), inducible ischemia (HR 1.76, 95% CI 1.28–2.41; *p* < 0.0001), resting LVEF (HR 0.96, 95% CI 0.95–0.97; *p* < 0.0001), and diabetes (HR 1.51, 95% CI 1.19–1.91; *p* < 0.0001).

To verify whether revascularization could affect these results, a Cox analysis was also conducted with the censoring of patients undergoing revascularization. Again, age, CFVR of LAD, inducible ischemia, resting LVEF, and diabetes were independently associated with mortality (Supplementary Material S4, Table S2).

RF algorithm (model and performance). The optimally tuned RF had 500 trees with 6 mtry and a node size of 5. The final performance achieved by the best-tuned model was an 80.22 C-index value. The RF is the leading performing algorithm in comparison with other classical MLs; the C-index for CTree is 76.5, GBM is 79, Coxnet is 79.3, and Xgboost is 76.6.

**Figure 2:**
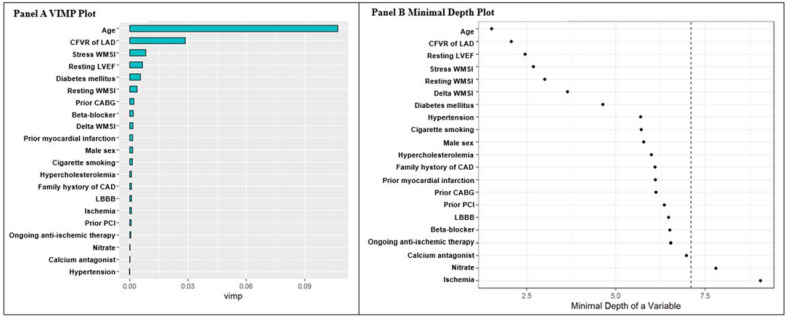
**Panel A:** Random forest VIMP plot. Bars are colored by a sign of VIMP; longer blue bars indicate more important variables. **Panel B:** minimal depth variables in rank order, most important at the top. The vertical dashed line indicates the maximal minimal depth for important variables. The mean of the minimal depth distribution is used as the threshold value for deciding whether a variable’s minimal depth value is small enough for the variable to be classified as strong.

Considering the VIMP metric, the leading predictors of survival were age, CFVR of LAD, stress WMSI, and resting LVEF (Figure 2, Panel A). The main predictors were the same considering the minimal depth measure. The mean of the minimal depth distribution was used as the threshold to exclude nitrate and ischemia as relevant features (Figure 2, Panel B).

A substantial agreement between the VIMP and minimal depth measure was observed, with the exception of gender and resting WMSI variables presenting a higher minimal depth rank order, and beta blocker and calcium antagonist features presenting a higher VIMP ranking (Supplementary Material S5, Figure S1).

**Figure 3:**
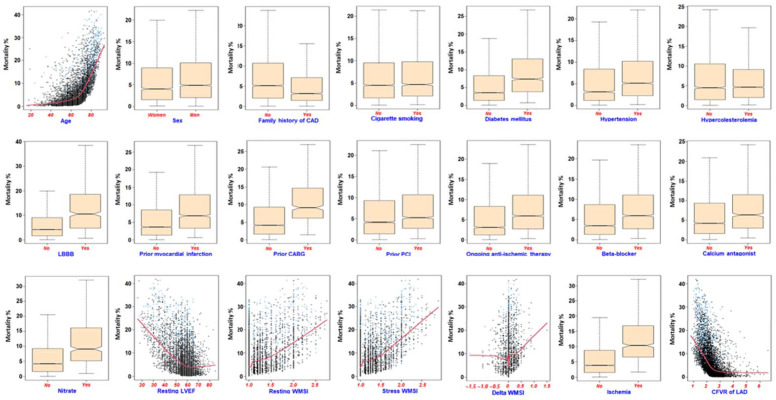
Variable dependence plot. Individual case predictions are marked with points. A less smooth curve indicates the trend as the variables increase with a shaded 95% confidence band. The mean of the minimal depth distribution is used as the threshold value for deciding whether a variable’s minimal depth value is small enough for the variable to be classified as strongPoints in blue correspond to events, black points are censored observations.

RF effect plot. The variable dependence plot (Figure 3) evidenced an increased mortality risk for prior CABG, beta blocker, diabetes, left bundle branch block, ischemia, and previous CABG. Moreover, the mortality risk was higher for patients aged more than 60 years, with a resting ejection fraction < 60%, resting WMSI, positive delta WMSI scores, and CFVR < 3 (Figure 3).

The partial (risk-adjusted) dependency plot evidenced the same pattern with less variability across modalities (for categorical variables) and data points for continuous variables (Supplementary Material S6, Figure S2).

The bootstrap C-index was also computed as an RF model. The achieved performance is lower (0.78) in comparison with the RF model (0.80); moreover, several convergence issues were identified during the iterations because conventional statistical models, in comparison with machine learning methods, are not tailored to handle high-dimensional data reporting a considerable number of features and collinearity issues [28].

Moreover, the Schoenfeld residuals test against the transformed time was computed. The global *p*-values < 0.001 indicate the violation of the proportionality assumption characterizing the Cox model. The RF model, instead, does not make any assumptions concerning the distributional form of the hazard [29].

**Figure 4:**
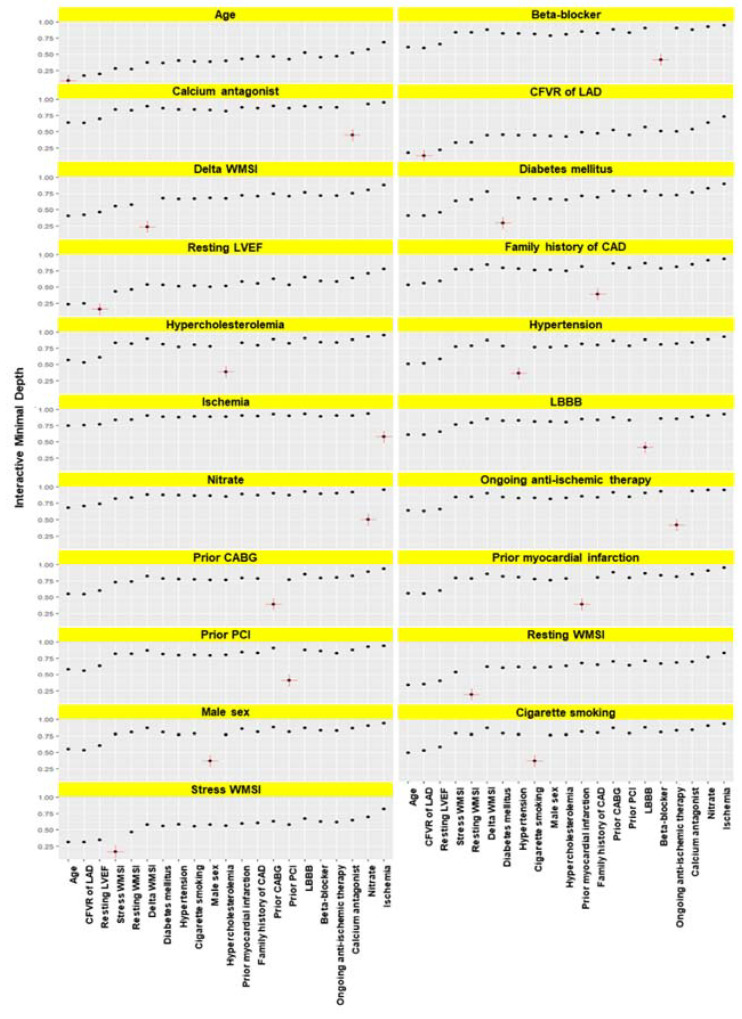
Minimal depth variable interactions. Reference variables are marked in red within each panel. Higher values indicate a lower reference variable.

The interaction plot identified a higher interaction effect across the variables previously identified as the most important such as age, CFVR of LAD, stress WMSI, and resting ejection fraction (Figure 4). 

The survival function can be easily obtained from an open-source web-based application after input of demographic, clinical, and SE data (https://r-ubesp.dctv.unipd.it/shiny/Dynamic/, accessed on 3 May 2022).

Concerning the internal validation, the OOB C-index performance computed internally via procedure A by comparing the true survival with the average OOB survival is 80.22. The RF median 10-fold cross-validated C-index statistic (procedure B) is, for the RF model, 0.79 (SD = 0.023). The Cox regression model demonstrated a slightly lower performance and higher variability between computations at various folds; the C-index measure is 0.75 (SD = 0.024). The C-index calculated on the external validation set is 0.81 and 0.78 for the Cox model.

## 4. Discussion

An ML approach combining simple clinical and SE variables can be used to predict the survival of patients undergoing SE with the dual assessment of RWMAs and CFVR (central illustration). These data confirm and expand previous evidence suggesting that RWMAs and CFVR can independently predict all-cause death, and their predictive value can be magnified when combined with simple clinical variables [3,8]. The identification of overall risk is central in initiating and titrating interventions, since the benefit of actionable interventions (initiation of statin or anti-platelet therapy, improved management of hypertension, etc.) is expected to be higher in the presence of higher overall risk. This risk is generally estimated on the basis of clinical variables that are clearly outperformed by SE variables combined with clinical variables.

There are also two relatively novel aspects that were not exposed by an outcome analysis involving SE data thus far. One important aspect is the linearly shaped relationship between WMSI and survival. Zero points mean that there are no changes during stress, with a normal or fixed (scar) response. Positive values for ischemia with higher values indicate more extensive and/or severe ischemia. In keeping with what is largely known, more extensive ischemia is associated with a higher risk. The new finding of the present study is that the slope of the relationship between risk and mortality is steadily growing and is steep. 

A second aspect relates to CFVR, since the linearly shaped curve associating CFVR and mortality started to rise below 3.0, well above the conventional diagnostic value of 2.0 usually adopted as an age- and sex-independent cutoff [30]. This finding can be considered less surprising, since it has been previously observed that the prognosis linked to CFVR is better stratified with a continuous rather than binary response, and patients in the highest quartile (>2.61) had 4-fold fewer chances of hard events per year than patients in the lowest quartile (<1.80) [31].

Study limitations.

All recruiting centers are from Italy, and therefore, the ethnical and geographic mix is limited; this may limit the generalizability of the algorithm. This issue will be likely solved with the ongoing stress echo 2030 study, applying this same approach to patients from four continents and 30+ recruiting countries.

The clinical application of the app is for scientific use only at this point, since further validation is needed, especially in diverse demographic groups with different stress modalities such as exercise or dobutamine, and especially considering that our data came from centers all accredited with joint reading sessions and quality control procedures before being allowed to enter the SE databank.

About 15% of individuals were excluded in the screening phase, which may pose significant selection biases; however, this is unavoidable in a study focused on cardiac functional stress testing with imaging methods, which is neither clinically appropriate nor technically feasible in all patients with all parameters.

All-cause death is a methodologically robust and clinically meaningful end-point [32], but the results of SE should more intuitively predict cardiovascular death, although both RWMAs [33] and CFVR [34] also predict cancer death, possibly due to the common biological and epidemiological roots of the two diseases [35]. In our study population, the mortality rate is 12% after a median follow-up of 3.4 years, which is not particularly high if we consider that all-cause death is the end-point, 40% of causes of death are from noncardiovascular causes in this population, and a 1 to 3% annual mortality rate from cardiac death is considered an intermediate risk in this population of stable patients referred to cardiac functional stress testing [1,33]. A family history of CAD and hypercholesterolemia were protective on mortality, which may appear surprising but is possibly explained by the more aggressive treatment of risk factors in these patients, including a more liberal use of stains that may exert a protective effect on mortality in a way largely independent from cholesterol levels. However, the structure of the data set did not allow us to correlate prognosis with initiation and duration and dose of drug interventions.

## 5. Conclusions

The present study shows the feasibility and usefulness of an ML approach for outcome prediction based on SE results in a large data set. The ML approach discovered interactions missed by the conventional Cox analysis. Within the adopted ML model, survival decreases constantly and steeply for ischemia values above 0 of delta (stress-rest) WMSI and below 3 of CFVR. Even mildly subnormal CFVR values impact mortality. An approach based on ML is also the methodological and conceptual platform necessary to handle even more complex data sets in the future, since comprehensive SE is now based on multiple parameters, much above the conventional step A of RWMAs embedded in the guidelines and step D (Doppler-based CFVR) included in the present analysis. The new letters are step B (for B-lines by lung ultrasound), step C (for cardiac reserve based on volumetric echocardiography), and step E based on the imaging-independent, electrocardiogram-based assessment of heart rate reserve, which is an index of cardiac autonomic balance. Each step shows independent and incremental value for predicting events [36] and survival [37]. Due to the largely automated analysis process involved, the ML approach can easily be scaled to larger databases with more comprehensive information, providing a user-friendly risk stratification tool available to all physicians.

## Figures and Tables

**Table 1:** Univariable prognostic predictors.

Variable	Alive	Death	Overall	HR	95% CI	*p*-Value
	(N = 6067)	(N = 814)	(N = 6881)			
Age (Years)	58/66/73	69/76/80	59/67/74	1.1	1.09, 1.11	<0.001
Gender: Female	41% (2489)	38% (312)	41% (2801)	-	-	-
Male	59% (3578)	62% (502)	59% (4080)	1.16	1.00, 1.33	0.044
Family history of CAD: No	71% (4286)	79% (642)	72% (4928)	-	-	-
Yes	29% (1781)	21% (172)	28% (1953)	0.6	0.51, 0.71	<0.001
Cigarette smoking: No	71% (4325)	73% (595)	71% (4920)	-	-	-
Yes	29% (1742)	27% (219)	29% (1961)	0.99	0.85, 1.15	0.89
Diabetes mellitus: No	77% (4672)	64% (521)	75% (5193)	-	-	-
Yes	23% (1395)	36% (293)	25% (1688)	1.77	1.53, 2.04	<0.001
Hypertension: No	33% (2026)	29% (240)	33% (2266)	-	-	-
Yes	67% (4041)	71% (574)	67% (4615)	1.29	1.11, 1.50	<0.001
Hypercholesterolemia: No	44% (2654)	50% (406)	44% (3060)	-	-	-
Yes	56% (3413)	50% (408)	56% (3821)	0.87	0.76, 0.99	0.041
LBBB: No	93% (5650)	88% (719)	93% (6369)	-	-	-
Yes	7% (417)	12% (95)	7% (512)	2.05	1.65, 2.54	<0.001
Prior myocardial infarction: No	76% (4617)	69% (563)	75% (5180)	-	-	-
Yes	24% (1450)	31% (251)	25% (1701)	1.49	1.28, 1.73	<0.001
Prior CABG: No	94% (5731)	89% (721)	94% (6452)	-	-	-
Yes	6% (336)	11% (93)	6% (429)	1.81	1.46, 2.25	<0.001
Prior PCI: No	74% (4511)	75% (608)	74% (5119)	-	-	-
Yes	26% (1556)	25% (206)	26% (1762)	1	0.85, 1.17	0.96
Ongoing anti-ischemic therapy: No	54% (3278)	53% (434)	54% (3712)	-	-	-
Yes	46% (2789)	47% (380)	46% (3169)	1.35	1.18, 1.56	<0.001
Beta blocker: No	62% (3765)	62% (504)	62% (4269)	-	-	-
Yes	38% (2302)	38% (310)	38% (2612)	1.31	1.14, 1.51	<0.001
Calcium antagonist: No	86% (5209)	86% (703)	86% (5912)	-	-	-
Yes	14% (858)	14% (111)	14% (969)	1.25	1.02, 1.52	0.035
Nitrate: No	94% (5691)	91% (743)	94% (6434)	-	-	-
Yes	6% (376)	9% (71)	6% (447)	1.64	1.29, 2.10	<0.001
Resting LVEF	55/60/62	50/58/60	54/60/62	0.95	0.95, 0.96	<0.001
Resting WMSI	1.0/1.0/1.1	1.0/1.0/1.4	1.0/1.0/1.1	3.2	2.70, 3.81	<0.001
Stress WMSI	1.0/1.0/1.2	1.0/1.0/1.4	1.0/1.0/1.2	1.57	1.47, 1.68	<0.001
Delta WMSI	0/0/0	0/0/0	0/0/0	2.66	1.47, 4.83	0.002
Ischemia: No	90% (5478)	89% (725)	90% (6203)	-	-	-
Yes	10% (589)	11% (89)	10% (678)	1.89	1.52, 2.36	<0.001
CFVR of LAD	2.0/2.3/2.7	1.6/2.0/2.3	2.0/2.3/2.6	0.29	0.25, 0.33	<0.001

Continuous data are reported as medians (I, III quartiles); categorical data are reported as a percentage and absolute frequencies. Univariable Cox regression hazard estimates are reported together with the 95% confidence intervals (CIs) and *p*-values. CAD = coronary artery disease; LBBB = left bundle branch block; CABG = coronary artery bypass grafting; PCI = percutaneous coronary intervention; LVEF = left ventricular ejection fraction; WMSI = wall motion score index; CFVR = coronary flow velocity reserve; LAD = left anterior descending artery.

## Data Availability

Data are available upon reasonable request to the authors.

## Supplementary Materials

The following supporting information can be downloaded at: https://www.mdpi.com/article/10.3390/jpm12091523/s1. Supplementary Material S1; Supplementary Material S2: Table S1: Descriptive statistics of the external validation cohort. The data have been reported as median, I, and III quartiles for continuous variables and as absolute and relative frequencies for the categorical variables. The Univariable Cox-regression Hazard Ratio (HR) with 95% con *p*-value has been also reported for the survival endpoint; Supplementary Material S3; Supplementary Material S4: Table S2: Univariable and multivariable predictors of mortality with censoring of patients undergoing revascularization; Supplementary Material S5: Figure S1: Minimal Depth and Vimp rankings. Points on the red dashed line are ranked equivalently, points below have higher VIMP, those above have higher minimal depth ranking. Variables are colored by the sign of the VIMP measure. Vertical dashed line indicates the maximal minimal depth for important variables. The mean of the minimal depth distribution is used as the threshold value for deciding whether a variable’s minimal depth value is small enough for the variable to be classified as strong; Supplementary Material S6: Figure S2: Partial dependence panels. Risk adjusted variable dependence for variables in minimal depth rank order.
