# Supplementary material for: Machine Learning Algorithms for Prediction of Survival by Stress Echocardiography in Chronic Coronary Syndromes
Source: J Pers Med. 2022 Sep 16;12(9):1523. doi: 10.3390/jpm12091523 (PMC9504503; doi:10.3390/jpm12091523)

Supplementary Material

Supplementary S1 Material

Importance Measures

Variable importance measure. The variable importance (VIMP) plot has been reported. The VIMP metric is the difference between out-of-bag prediction errors before and after variable removal within permutation. The importance of a specific variable is computed as the average increase in the out-of-bag error across all trees when the values of that variable are permuted (8). The input variable which causes the largest increase in error is considered to be the most important. A VIMP value close to zero indicates the variable does not contribute to the predictive accuracy of the model.

Minimal Depth interaction. The VIMP measure ranks the most important variables according to their impact on the predictive ability of the model. The Minimal Depth is an alternative metric assuming that the feature more impacting on the final prediction is the variable which most frequently split nodes nearest to the root node. The tree node levels are numbered based on their relative distance to the root node. The Minimal Depth measures are calculated by averaging the depth of the first split for each variable over all trees within the forest. The minimal depth of a variable is a proxy of the feature predictiveness; a smaller measure indicates a higher impact of the variable on the RF prediction (13).

RF effect plots. The RF predicted response dependency on covariates has been investigated by reporting the *variable dependence* and the *partial dependency* plot.

Variable dependence plots show the predicted response as a function of a covariate of interest. Each predicted point is an individual observation, dependent on the full combination of all other covariates not only on the covariate of interest (14).

Partial dependence plots are a risk-adjusted alternative to variable dependence. Partial plots are generated by averaging a set of predictions for each observation in the training set at a specific value of the covariate $X=x$. The process has been repeated for a sequence of covariate values to generate the estimated points to be reported on the partial dependence plot (15).

Supplementary S2 Material

Table S1. Descriptive statistics of the external validation cohort. The data have been reported as median, I, and III quartiles for continuous variables and as absolute and relative frequencies for the categorical variables. The Univariable Cox-regression Hazard Ratio (HR) with 95% con P-value has been also reported for the survival endpoint

**Variable**	**N**	**Alive (N=841)**	**Death (N=161)**	**Overall (N=1002)**	**HR**	**95% CI**	**P-value**
**Age** (Years)	1002	58/66/73	70/75/81	59/68/74	1.1	1.08, 1.13	<0.001
**Gender**: Female	1002	39% (328)	34% (55)	38% (383)	-	-	-
Male	-	61% (513)	66% (106)	62% (619)	1.22	0.88, 1.69	0.23
**Family history of CAD**: No	1002	68% (571)	76% (122)	69% (693)	-	-	-
Yes	-	32% (270)	24% (39)	31% (309)	0.67	0.47, 0.97	0.03
**Cigarette smoking**: No	1002	79% (665)	86% (139)	80% (804)	-	-	-
Yes	-	21% (176)	13% (22)	20% (198)	0.61	0.39, 0.96	0.03
**Diabetes mellitus**: No	1002	79% (662)	70% (112)	77% (774)	-	-	-
Yes	-	21% (179)	30% (49)	23% (228)	1.54	1.10, 2.15	0.01
**Hypertension**: No	1002	35% (291)	32% (51)	34% (342)	-	-	-
Yes	-	65% (550)	68% (110)	66% (660)	1.09	0.78, 1.52	0.61
**Hypercholesterolemia**: No	1002	46% (386)	50% (70)	46% (456)	-	-	-
Yes	-	54% (454)	44% (91)	54% (545)	1.04	0.76, 1.43	0.79
**LBBB**: No	1002	98% (823)	99% (159)	98% (982)	-	-	-
Yes	-	2% (18)	1% (2)	2% (20)	0.60	0.15, 2.44	0.47
**Prior myocardial infarction**: No	1002	82% (687)	69% (111)	80% (798)	-	-	-
Yes	-	18% (154)	31% (50)	20% (204)	1.84	1.32, 2.57	<0.001
**Prior CABG**: No	1002	95% (802)	88% (142)	94% (944)	-	-	-
Yes	-	5% (39)	12% (19)	6% (58)	2.33	1.45, 3.77	<0.001
**Prior PCI**: No	1002	76% (639)	72% (116)	75% (755)	-	-	-
Yes	-	24% (202)	28% (45)	25% (247)	1.16	0.82, 1.63	0.40
**Ongoing anti-ischemic therapy**: No	1002	73% (612)	70% (113)	72% (75)	-	-	-
Yes	-	27% (229)	30% (48)	28% (277)	1.11	0.79, 1.56	0.54
**Beta blocker**: No	1002	75% (629)	72% (116)	74% (745)	-	-	-
Yes	-	25% (212)	28% (45)	26% (257)	1.13	0.80, 1.60	0.48
**Calcium antagonist**: No	1002	98% (824)	98% (158)	98% (982)	-	-	-
Yes	-	2% (17)	2% (3)	2% (20)	0.87	0.28, 2.73	0.81
**Nitrate**: No	1002	99% (835)	99% (159)	99% (994)	-	-	-
Yes	-	1% (6)	1% (2)	1% (80)	1.48	0.37, 5.99	0.58
**Resting LVEF**	1002	60/60/60	45/60/60	60/60/60	0.96	0.95, 0.98	<0.001
**Resting WMSI**	1002	1.0/1.0/1.0	1.0/1.0/1.1	1.0/1.0/1.0	3.29	1.70, 6.36	<0.001
**Stress WMSI**	1002	1.0/1.0/1.1	1.0/1.0/1.2	1.0/1.0/1.1	3.60	2.05, 6.35	<0.001
**Delta WMSI**	1002	0/0/0	0/0/0	0/0/0	10.04	2.32, 46.5	0.002
**Ischemia**: No	1002	84% (705)	76% (122)	82% (827)	-	-	-
Yes	-	16% (136)	24% (39)	18% (175)	1.52	1.06, 2.18	0.02
**CFVR of LAD**	1002	1.9/2.1/2.3	1.5/1.7/1.9	1.8/2.0/2.3	0.08	0.05, 0.13	<0.001

Abbreviations as in Table1.

Supplementary S3 Material

Cardiovascular Imaging-Related Machine Learning Evaluation (PRIME) requirements

1. Designing the study plan: describing the need for the application of ML to the data set (see introduction); and providing summary statistics of baseline data (see after: Results, Table 1 and Table 2);
2. Data standardization: we used a well-established data set with zero fraction of missing values and we included outliers since data were controlled upstream to entering the database with reading harmonization and readers certification;
3. Model selection process: since the primary goal of the analysis was classification (outcome, live or dead), we selected a supervised learning method based on Random Forest after the hyperparameter tuning, and we also provided a direct comparison of the ML with the simpler Cox model;
4. Description of data used for training, validation, and testing: they are reported in the methods section
5. Model evaluation: the accuracy of the model was reported in a way familiar to the clinician as a survival curve from freely downloadable app;
6. Limitations, biases and alternatives: they are reported in the discussion (study limitations, see after)

Supplementary S4 Material

Table S2. Univariable and multivariable predictors of mortality with censoring of patients undergoing revascularization

	Univariable analysis		Multivariable analysis	
	**HR (95% CI)**	**p value**	**HR (95% CI)**	**p value**
Age (yrs)	1.08 (1.07-1.10)	<0.0001	1.08 (1.06-1.09)	<0.0001
Gender (male)	0.84 (0.64-1.10)	0.20		
Family history of CAD	0.68 (0.50-0.92)	0.01		
Cigarette smoking	0.93 (0.72-1.20)	0.56		
Diabetes mellitus	1.76 (1.37-2.25)	<0.0001	1.46 (1.13-1.88)	0.003
Hypertension	1.00 (0.81-1.34)	0.75		
Hypercholesterolemia	0.75 (0.59-0.95)	0.02		
LBBB	1.95 (1.48-2.58)	<0.0001		
Prior myocardial infarction	0.85 (0.67-1.08)	0.19		
Prior CABG	1.27 (0.93-1.74)	0.12		
Prior PCI	0.63 (0.49-0.81)	<0.0001		
Ongoing anti-ischemic therapy	0.94 (0.74-1.20)	0.61		
β-blocker	0.89 (0.70-1.13)	0.35		
Calcium antagonist	1.28 (0.92-1.78)	0.15		
Nitrate	1.43 (1.00-2.05)	0.05		
Resting LVEF	0.96 (0.95-0.97)	<0.0001	0.97 (0.95-0.98)	<0.0001
Resting WMSI	2.61 (1.94-3.52)	<0.0001		
Stress WMSI	3.77 (2.79-5.11)	<0.0001		
Delta WMSI	6.47 (2.80-14.93)	<0.0001		
Ischemia	2.24 (1.64-3.07)	<0.0001	2.25 (1.61-3.17)	<0.0001
CFVR of LAD	0.30 (0.23-0.39)	<0.0001	0.57 (0.43-0.75)	<0.0001

HR= hazard ratio; CI = confidence interval.

Other abbreviations as in Table 1.

Supplementary S5 Material

Figure S1. Minimal Depth and Vimp rankings. Points on the red dashed line are ranked equivalently, points below have higher VIMP, those above have higher minimal depth ranking. Variables are colored by the sign of the VIMP measure. Vertical dashed line indicates the maximal minimal depth for important variables. The mean of the minimal depth distribution is used as the threshold value for deciding whether a variable's minimal depth value is small enough for the variable to be classified as strong.

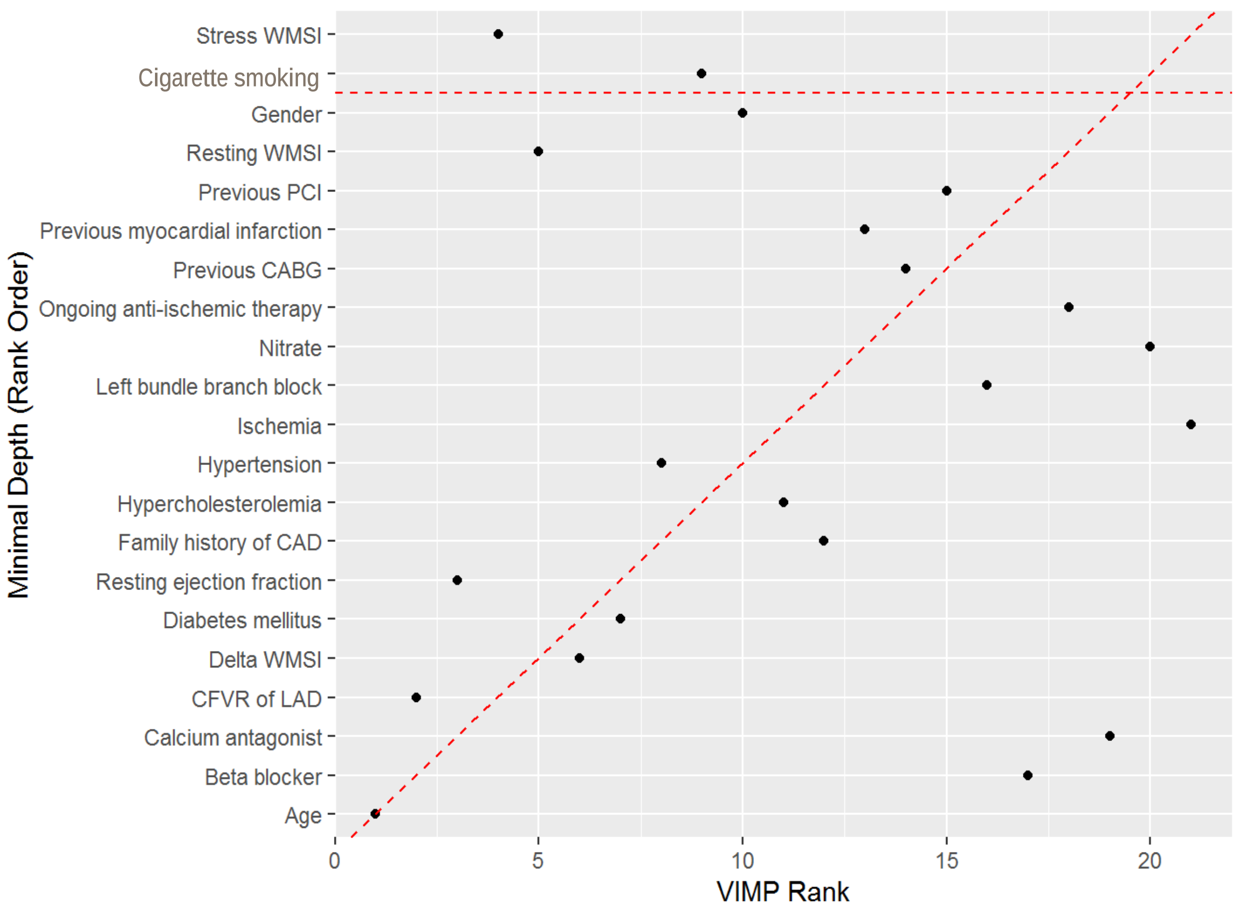

Supplementary S6 Material

Figure S2. Partial dependence panels. Risk adjusted variable dependence for variables in minimal depth rank order.

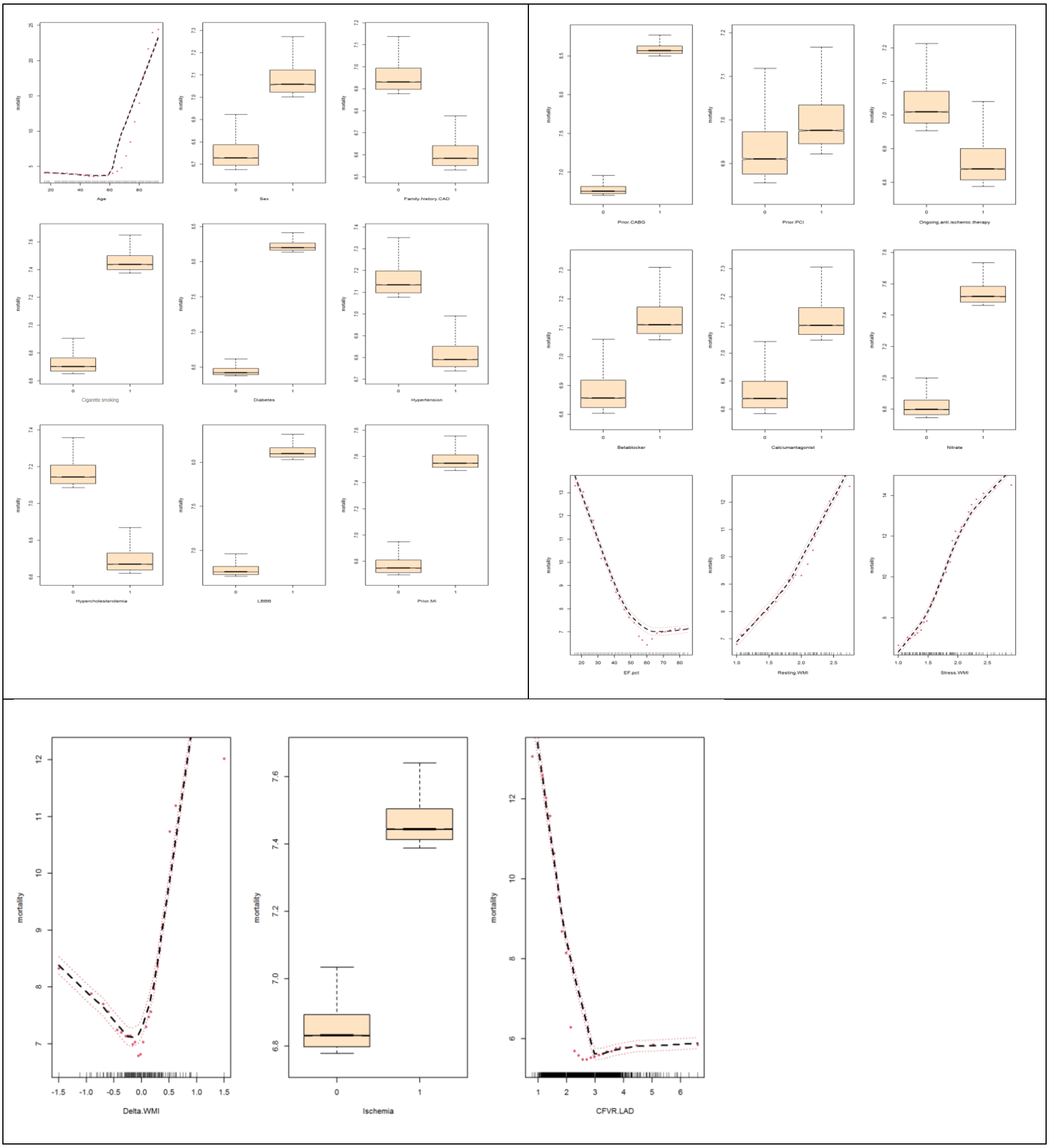

Supplement: Supplementary file 1 [file jpm-12-01523-s001.zip › jpm-1878956-supplementary.pdf]
